# A high-resolution view of the heterogeneous aging endothelium

**DOI:** 10.1007/s10456-023-09904-6

**Published:** 2024-02-07

**Authors:** Sarah Dobner, Fanni Tóth, Laura P. M. H. de Rooij

**Affiliations:** The CeMM Research Center for Molecular Medicine of the Austrian Academy of Sciences, Vienna, Austria

**Keywords:** Endothelial cell heterogeneity, Single-cell RNA-sequencing, Transcriptomics, Vascular aging

## Abstract

Vascular endothelial cell (EC) aging has a strong impact on tissue perfusion and overall cardiovascular health. While studies confined to the investigation of aging-associated vascular readouts in one or a few tissues have already drastically expanded our understanding of EC aging, single-cell omics and other high-resolution profiling technologies have started to illuminate the intricate molecular changes underlying endothelial aging across diverse tissues and vascular beds at scale. In this review, we provide an overview of recent insights into the heterogeneous adaptations of the aging vascular endothelium. We address critical questions regarding tissue-specific and universal responses of the endothelium to the aging process, EC turnover dynamics throughout lifespan, and the differential susceptibility of ECs to acquiring aging-associated traits. In doing so, we underscore the transformative potential of single-cell approaches in advancing our comprehension of endothelial aging, essential to foster the development of future innovative therapeutic strategies for aging-associated vascular conditions.

## Introduction

As we age, the functionality of our major organs declines. Aging-associated phenotypes in all cell types of the body emerge as a result of cellular and tissue microenvironment changes, driven by various genetic and environmental stressors. Numerous hallmarks of the aging process have been identified over the years, including genomic instability, telomere attrition, mitochondrial dysfunction, cellular senescence, and stem cell depletion [1]. Cardiovascular health is particularly impacted by these manifestations of aging, due to (at least in part) the compromised health of endothelial cells (ECs). ECs form the inner lining of our blood vessels and play a crucial role in safeguarding and maintaining the proper function of all organs and physiological systems in our body by acting as barriers, filters, homeostatic regulators, and mediators of immune cell trafficking and cell–cell communication. The aging process affects the endothelium in countless ways, for instance via remodeling of the vascular wall, oxidative and nitrosative stress, and impairment of angiogenesis (we refer to Ungvari et al*.* [2] and Brandes, Fleming and Busse [3] for in-depth reviews on this topic).

To gain deeper insights into the molecular changes associated with endothelial aging, it is imperative to understand the fundamentals of EC behavior, molecular circuitries, and interactions across lifespan as well as in aging-associated disease conditions. Numerous methodologies and models have been used over the years to, typically, profile one or a handful of aging-associated features of the endothelium at a time, usually in ECs derived from one particular tissue or vascular bed (i.e., the sum of the blood vessels supplying an organ or region). For the longest time, technological challenges have however hindered our ability for a highly precise and comprehensive investigation of EC biology at scale, and in an untargeted fashion. Fortunately, recent advancements in single-cell omics have paved the way for creating detailed tissue atlases and examining single cells in health and disease. In that light, numerous atlasing efforts have drastically increased our understanding of the heterogeneous vascular endothelium, now widely appreciated to exhibit distinct gene and protein expression profiles based on its tissue of origin and disease status [4–12]. Our knowledge of the precise molecular changes in response to age-related challenges in ECs from individual organs, however, remains poorly understood. Obtaining a high-resolution view of the aging endothelium, by exploiting single-cell technologies, will be imperative for (i) the identification of specific EC subtypes and gene expression programs influenced by the aging process, (ii) the unraveling of mechanisms underlying the initiation and progression of age-related EC decline, (iii) the definition of new markers indicative of various stages of vascular lifespan, and, ultimately, (iv) the discovery and development of innovative molecular, gene, and EC-centered therapies for rejuvenation and/or treatment of age-related (vascular) diseases.

While we are still far from achieving those goals, the number of studies exploring aging of tissues using single-cell omics is steadily expanding, and increasingly incorporating the vascular endothelium. Here, we provide an overview of the current state of the literature regarding the heterogeneous adaptations of the vascular endothelium over the course of aging. We highlight findings from single-cell transcriptomics studies, complemented with high-resolution insights obtained using alternative methods, to shed light on pressing, emerging questions in the field of vascular aging. For instance, does the process of aging affect ECs in different tissues or vascular beds in a similar way? Is there a pan-EC aging phenotype or signature across tissues? What is the true age of ECs in our body, and do all ECs exhibit a similar degree of turnover? Are ECs more or less prone to acquire aging-associated phenotypes (e.g., senescence, apoptosis) in comparison to other cell types? Lastly, we discuss the future perspectives and challenges ahead in uncovering vascular aging-associated endothelial heterogeneity.

### EC heterogeneity and aging in the single-cell era

**Fig. 1:**
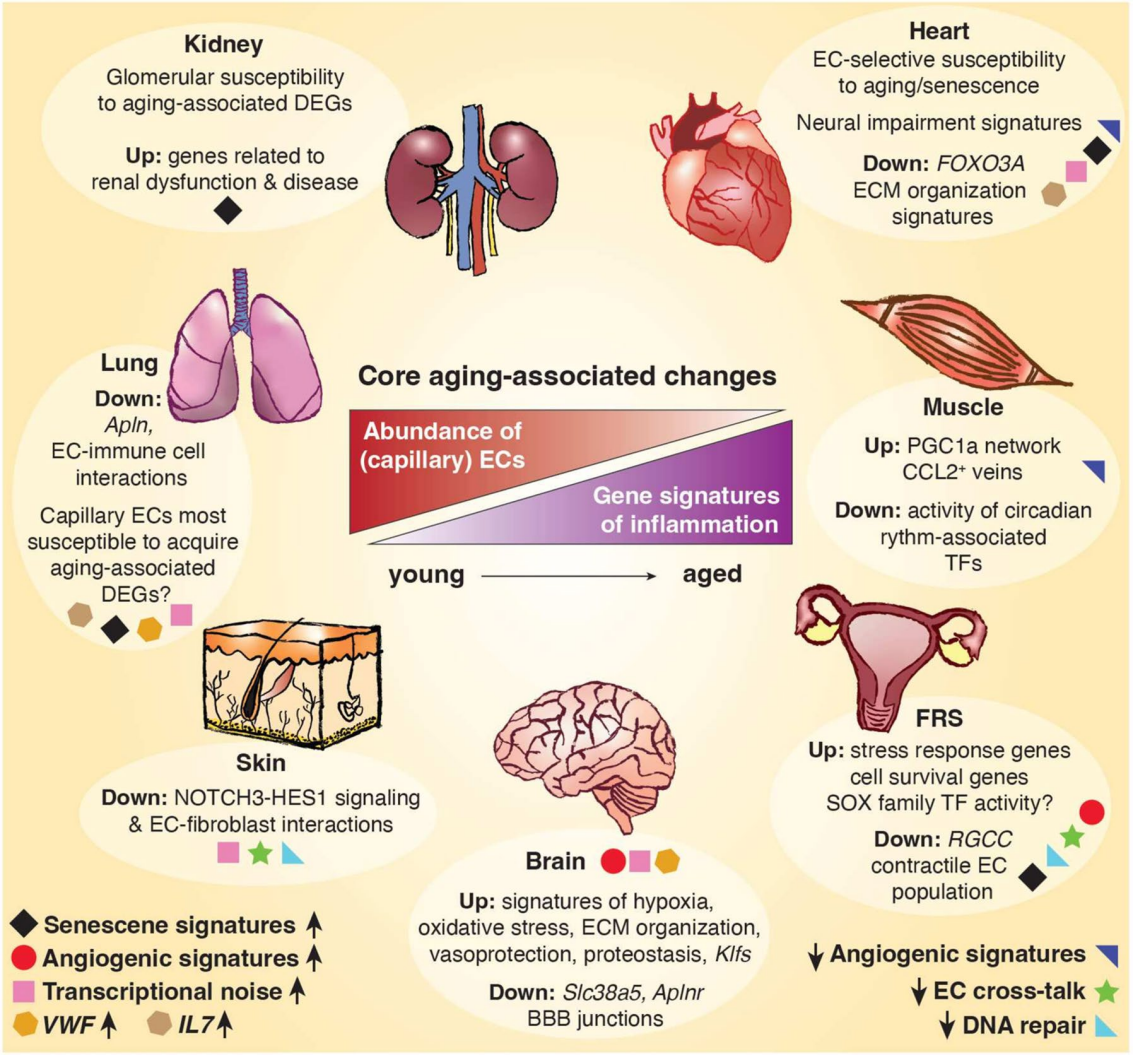
Global and tissue-specific changes of the healthy aging endothelium. Middle: Core aging-associated changes in the endothelial landscape, identified in single-cell transcriptomics studies across different tissues (human and/or primate and/or mouse), include a decreased abundance of ECs (in comparison to other cell types) with increasing age, typically via a reduction in capillary/microvascular subtype abundance. Gene signatures associated with immunoregulatory processes and inflammation are generally increased in aging ECs across organs and tissues. Tissue-specific changes of the endothelium with increasing age are indicated in circled boxes next to each tissue. Phenotypes shared among two–three tissues are indicated at the bottom, including elevated expression of senescence-associated gene signatures (FRS, heart, kidney, lung), either a reduced (muscle, heart) or increased (brain, FRS) expression of angiogenic gene signatures, reduced expression of DNA repair signatures (skin, FRS), fewer interactions between ECs and other cell types (skin, FRS), increased transcriptional noise (brain, heart, lung, skin), and increased expression of *VWF* (lung, brain) or *IL7* (heart, lung), as specified for each tissue by colored icons. EC = endothelial cell; ECM = extracellular matrix; DEG = differentially expressed gene; FRS = female reproductive system; Klfs = Krüppel-like factors; TF = transcription factor

Comprehensive atlasing efforts dedicated to exploring the endothelial transcriptome across tissues and ages are yet to be conducted. Nevertheless, recent endeavors have started to reveal the first insights into diverse responses of ECs and their subtypes to the aging process in individual organs and tissues. In the subsequent sections, we will highlight the reported findings thus far for several tissue vasculatures. Across studies, “core” aging-associated changes of the endothelium include the loss of microvascular marker gene expression and a reduced abundance of (capillary/microvascular) ECs, as well as increased expression of inflammation and immunoregulatory gene signatures. These common changes are accompanied by a wide range of tissue-specific features of the aging endothelium, which we summarize in Fig. 1 and Table 1.

**Table 1:** Common and tissue-specific changes in the healthy aging endothelium transcriptome landscape

	Transcriptional changes (up) ↑	Transcriptional changes (down) ↓	EC population changes	EC Turnover	Possible functional consequences	References
Brain	Signatures of: **Angiogenesis**, ECM organization, Glycolysis, Hypoxia, **Inflammation,** **IFN-signaling,** Krüppel-like factors, Oxidative stress, Proteostasis, Vasoprotection **Transcriptional noise** ***Vwf* expression**	Signatures of: Glycolysis and energy metabolism, cell–cell junctions *Slc38a5* expression *Aplnr* expression	Mixed findings: either no change in EC subtype abundance, or a loss of capillary-venous ECs Slight increase in number of ECs with senescence signature Capillary ECs most susceptible to aging-associated DEGs	X	Increased BBB permeability Reduced blood flow Neuronal death	[16, 26–31, 39, 43, 123]
FRS	Signatures of: **Angiogenesis (VEGF)**, Apoptosis, Cell cycle, Cellular survival, **Immunoregulation,** **Senescence**, Stress response *SOX* TF activity	Signatures of: **DNA repair**, Proteasome, Metabolism *RGCC* expression **Cross-talk** (Angiogenic, (HGF, ANGPTL, EDN)	*FLNA* + *ACTG2* + Contractile EC population Potential **decrease of capillary clusters** Decrease in vascular and lymphatic ECs		Hampered blood flow and vascularization	[39, 97–100]
Heart	Signatures of: Atherosclerosis, Neuronal impairment (*Sema3a*), **Senescence** ***IL7* expression** **Transcriptional noise**	Signatures of: **Angiogenesis**, ECM organization *FOXO3A* expression	**Decreased abundance of capillary clusters** Strong susceptibility to aging (coronary artery EC and capillary EC)	✓	Sympathetic denervation Potential reduction of vascular homeostasis and repair	[39, 73, 80–84, 127]
Kidney	Signatures of: **Inflammation,** **IFN-signaling,** **Senescence** (esp. glomerular ECs)		**Decreased abundance of capillary clusters** Glomerular ECs susceptible to aging-associated DEGs		Renal dysfunction, kidney disease	[39, 72, 80, 110, 136]
Lung	Signatures of:** Inflammation,** **IFN-signaling,** **Senescence** Expression of: ***Vwf****, NFKB1, HIF1A, ****IL7****, Il1b* **Cross-talk** (ECs & immune cells) **Transcriptional noise**	*Apln* expression	Aging-associated changes mostly detected in capillaries	✓ (general capillary ECs)	Potential impairment of vascular repair after injury (IPF, COPD, COVID-19)	[67, 70–74, 77]
Muscle	Signatures of: **Inflammation** **Immunoregulation (*Rfx5, Nfat5)***	Circadian rhythm regulation **Angiogenic signatures**	Increased abundance of *CCL2*^+^ veins **Decreased abundance of capillary clusters** Decreased abundance of arterial clusters		Vascular barrier integrity Potential impairment of skeletal muscle mass and function	[27, 39, 55, 56, 60]
Skin	**Transcriptional noise**	Signatures of: **DNA repair** **Cross-talk** (EC-fibroblast, NOTCH3-HES1 axis)	**Decreased abundance of capillary clusters**		Decreased repair potential, vascular stiffening	[93, 94]

Common transcriptomic changes shared between multiple tissues are indicated in bold

*EC* endothelial cell; *ECM* extracellular matrix; *BBB* blood–brain barrier; *FRS* female reproductive system; *TF* transcription factor; *DEG* differentially expressed gene; *IPF* idiopathic pulmonary fibrosis; *COPD* chronic obstructive pulmonary disorder; *SASP* senescence associated secretory phenotype

### Brain

Brain endothelial cells (BECs) are key components of the cerebral microvascular system, which provides a continuous supply of nutrients and oxygen to fulfill the high energy requirements of the brain [13]. As the primary cell type of the blood–brain barrier (BBB) and neurovascular unit, BECs have an essential role in the regulation of molecular transport in and out of the brain, protection of the brain from pathogens, and in the regulation of cerebral blood flow [14–16]. Representing one of the very first tissues for which the endothelium was profiled at single-cell resolution [17], we now have an in-depth understanding of endothelial arteriovenous zonation and the associated EC subtypes and gene expression patterns in the mouse and human brain [18, 19]. Aging has been associated with impaired brain function and an increased susceptibility to cognitive decline and neurodegenerative disease [20]. The age-related dysfunction of BECs contributes to deterioration of the cerebral microvascular system, and is responsible for increased BBB permeability [15, 21, 22], decreased cerebral blood flow [23], and impaired neurovascular coupling [13, 24], all of which are hallmarks of both normal aging and neurodegenerative diseases [25, 26].

#### Healthy aging

A wide range of single-cell transcriptomics studies of young versus aged disease-free mouse brains revealed endothelial aging-associated transcriptional profiles [16, 26–31]. While some of these studies report that the overall abundance of ECs, or their arteriovenous zonation patterns, were not changed between young and aged animals [16, 29, 31], others reported a decrease in capillary-venous ECs (i.e., EC subtype with mixed distribution of both capillary and venous marker genes) in brain tissue from old animals [30]. In terms of aging-associated gene expression changes, a common finding in these studies was a typical increase in expression for the majority of differentially expressed genes (DEGs) upon aging, rather than a decrease [16, 30]. Commonly upregulated genes in aged BECs include those related to inflammation and the immune response (e.g., *Vcam1, B2m, Cxcl12, Oas1*) and members of the interferon (IFN) signaling pathway (e.g., *Stat1, Bst2, Ifnar1*, *Gbp6* [16, 29, 31, 32]), in line with chronic inflammation as a well-established hallmark of aging [1]. These changes may be associated with age-dependent BBB dysfunction, as increased endothelial expression of pro-inflammatory cytokines has been linked with reduced expression of EC tight junction proteins [33] and BBB permeability [34]. Findings of decreased expression of classical BBB junction genes (e.g., *Ocln, Cldn5*) [30] in aged BECs are in line with this. In some studies, aged BECs also commonly exhibited upregulation of genes related to the hypoxia response (e.g., *Hif1a, Ldha, Aldoa*), and genes involved in oxidative stress pathways (e.g., *Sod1, Alpl*, *Apoe*), indicating an increased cellular stress response [16, 28]. Increased oxidative stress in BECs has been associated with age-induced impairment of the cerebral microvascular system and neurovascular uncoupling [13, 24, 35], age-related alterations of vascular reactivity [36] and reduced pericyte coverage of cerebral microvessels [37, 38], which could ultimately contribute to neuronal cell death [15]. Another common finding was the age-related upregulation of *Vwf*, a key regulator of hemostatic control, various Krüppel-like factors (e.g., *Klf2, Klf4, Klf6*), important for vasoprotection and response to injury, and genes involved in cell adhesion and/or extracellular matrix (ECM) organization (e.g., *Adamts1, Itga6, Cyr61*) [16, 28, 31]. Angiogenic signaling pathways, such as insulin-like growth factor-1 receptor (IGF-1R) , vascular endothelial growth factor (VEGF) and transforming growth factor beta (TGF-β),were also upregulated in aged BECs, indicated by the upregulation of genes such as *Igf1r*, *Kdr*, *Flt1*, *Edn1*, *Eng*, *Id1*, *Bmpr2*, and *Acvrl1* [16, 28, 30, 31]. These findings are in contrast with the well-known phenomenon of capillary rarefaction in aging tissues [39], including the brain [40], suggesting that these genes may be activated in a counteracting attempt to restore the lost vascular density. Besides enhancing angiogenesis, VEGF signaling has however also been associated with BBB disruption [41, 42]. Aged BECs furthermore showed an upregulation of glycolytic genes (e.g., *Pkm*, *Aldoa*, *Ldha*)*,* known to be important for angiogenesis, and which could indicate a shift toward glycolytic metabolism [16]. Altered metabolism is a well-known characteristic of EC aging and senescence (Box 1). Caution is nevertheless warranted, as Zhao and colleagues reported a selectively decreased expression for several metabolic genes involved in glucose/energy metabolism and ATP synthesis in aged brain capillaries [30]. Furthermore, Jin and colleagues reported decreased levels of *Aplnr* in aged mouse brain ECs, potentially impairing angiogenesis in healthy aging and after stroke [43]. BECs additionally exhibited an age-associated increase in protein synthesis, as demonstrated by an enrichment of genes involved in the proteostasis pathway [28] and the upregulation of ribosomal subunit genes, such as *Rpl37, Rps20*, and *Rps21* [16, 31]. A slight age-associated increase of senescent BECs was furthermore reported in a scRNA-seq study of the mouse brain [26]. The accumulation of senescent ECs has been directly connected to BBB dysfunction in vitro and in vivo [44], at least in part via induction of the senescence-associated secretory phenotype [15], and the dysregulation of nitric oxide signaling in BECs [45].

Of note, age-related changes of the BEC transcriptomic profile were reversible by infusion with plasma derived from young mice [16] or by heterochronic parabiosis (a surgical procedure where young and old mice are joined together to share a common circulatory system) [28]. Here, capillary BECs were found to be the most susceptible cell type in the mouse brain to the aging or rejuvenating effects of old and young blood or plasma, respectively [16, 28]. Moreover, exercise has been shown to exert beneficial effects on brain microvascular perfusion [46, 47], and single-cell profiling revealed a putative endothelial-specific role in this phenomenon [27]. Precisely, subventricular zone cell types in the mouse brain were shown to interact with ECs via TWEAK signaling and this interaction was lost in aged mice, likely driven by a loss of *Tnfrsf12a* expression in ECs. Exercise was associated with the restoration of *Tnfrsf12a* expression in ECs, suggesting this signaling axis to be at least one of the underlying drivers of the beneficial effect of exercise on health of the brain vascular endothelium [27]. Lastly, glucagon-like peptide-1 receptor agonist treatment (known to alleviate various cellular phenotypes of aging) was also shown to improve BBB integrity and reverse, at least in part, aging-associated gene expression changes in brain ECs of aged mice [30]. This again showcases the adaptable nature of aging-related gene expression changes in the aged brain endothelium.

#### Aging-associated disease

Based on recent scRNA-seq studies of Alzheimer’s disease (AD) and vascular dementia (VaD) in humans and mice, some of the genes that were upregulated in healthy aged BECs were also upregulated in AD- or VaD-associated ECs (e.g., *APOE, ALPL, B2M, HSPA4* and *VWF*) [18, 48, 49]. Moreover, expression of the amino acid transporter *Slc38a5*, a finding unique to aged BECs in the mouse (compared to other aging tissue ECs) [31], was confirmed to be decreased in human brain tumors and vascular malformations as well [50], indicating a possible link between the transcriptomic profile of age-related brain diseases and aging. This transporter is predominantly expressed in ECs of a capillary(-venous) nature in the human brain [51], possibly in line with the increased susceptibility of murine microvascular BECs to the aging process.

### Muscle

Skeletal muscle is typically highly vascularized, yet vascular density is known to be adaptable and dependent on the energy demands of muscle tissue [52]. Exercise rapidly stimulates vascularization in the muscle, and scRNA-seq in murine muscle tissue revealed the presence of distinct ATF3/4^+^ and ATF3/4^low^ capillary ECs with high and low angiogenic potential, respectively [53]. Loss of muscle mass and strength is common in the elderly and associated with increased frailty and mortality, and single-cell studies are starting to shed light on intricate changes to muscle EC phenotypes associated with aging (Fig. 1, Table 1). In time, findings from such studies will hopefully contribute to unraveling whether aging for instance causes a decline in EC function and angiogenesis in muscle tissue, which thus far remains a matter of debate [54].

#### Healthy aging

In a scRNA-seq analysis of young and aged healthy mice, altered immunoregulation and an increased transcriptional inflammatory score was evident in aged muscle ECs [27]. Physical exercise reduced this inflammatory phenotype in both young and aged mice, albeit at a much lower magnitude in aged mice compared to young animals [27]. In a single-cell and -nucleus transcriptome atlas of healthy human skeletal muscle across ages, another layer of evidence for pro-inflammatory endothelial phenotypes has emerged. While arterial and capillary ECs were shown to be strongly depleted in aged individuals, a drastic increase in veins expressing the proinflammatory cytokine CCL2 was observed in aged muscle. In line with this, CCL2^+^ veins and several other microenvironmental subpopulations of cells enriched for CCL2 (e.g., fibroblasts, smooth muscle cells, pericytes) were predicted to recruit different types of immune cells to muscle tissue in aged mice via its receptors (CCR2, CCR10) [55], again suggesting an overall increased pro-inflammatory environment in the aging muscular vasculature. Lastly, besides an upregulation of several immunoregulatory transcription factor (TF) activities (e.g., *Rfx5, Nfat5*), aging muscle ECs in the mouse were intriguingly shown to exhibit decreased activity of *Arntl* and *Clock* TFs [56], which are involved in regulating the circadian clock. Of note, their activity was most severely affected in the muscle vasculature compared to all other cell types in various aged murine organs. Accumulating evidence poses circadian rhythms as a critical regulator of skeletal muscle mass and function [57, 58], and circadian clock genes are known to markedly change with advancing age [59, 60]. These results may nevertheless stress an underappreciated role of the muscle endothelial compartment in this process.

#### Aging-associated disease

Of particular interest in the context of muscle aging-related pathology are sarcopenia (aging-associated loss of muscle mass) and cachexia (loss of weight due to an underlying sickness). EC dysfunction has been repeatedly shown to be associated with increased phenotypes of frailty and sarcopenia [61]. Moreover, recent findings indicate that skeletal muscle vascular attrition precedes cachexia in a mouse model of pancreatic ductal adenocarcinoma, and is associated with increased vascular leakiness and immune cell infiltration [62]. In line with muscle EC phenotypes in healthy aging, scRNA-seq revealed that muscles in cachexic mice harbor a unique EC subtype with a gene expression signature indicative of decreased angiogenesis and increased immunoregulation. Additionally, multiple cofactors of the transcriptional coactivator PGC1α were found expressed in this EC subtype, which were functionally validated to be important for maintenance of muscle vascular barrier integrity [62].

### Lung

Overall effects of EC aging in the lung have been presented in much detail in several previous reviews, that discuss mechanical influences on the aging vasculature [63, 64], as well as the role of ECs in different age-related pulmonary diseases [64, 65]. Single-cell studies have however added incremental complexity to our knowledge of pulmonary endothelial heterogeneity, as several specialized lung EC populations have been uncovered at transcriptional resolution over the recent years. For instance, the endothelium lining vessels surrounding the bronchi and major airways can be specifically identified by expression of markers including *COL15A1, ZNF385B* and *SPRY1* [9, 66]. Moreover, the lung harbors distinct capillary subtypes, including “general capillary ECs,” expressing *BTNL9*, *FCN3, GPIHBP1* and *CD36* (among others) [9], and the more specialized “aerocytes,” large capillary ECs expressing *CA4, EDNRB*, and *HPGD* (among others), responsible for gas exchange on the epithelial-alveoli interface [9, 67].

#### Regenerative capacities of specialized pulmonary EC subtypes

In mice, Car4^+^ capillaries (presumably aerocytes) were found to drastically expand after injury [68], likely in a VEGF-A dependent fashion regulated by AT1 epithelial cells [69]. However, independent studies have poised general capillary ECs as a potential endothelial stem cell population, giving rise to aerocytes during the repair phase after injury [67, 70]. During early phases of recovery (day 3 after injury), a subtype of general capillary ECs appeared and restored EC populations in the mouse lung [70]. Trajectory analyses revealed an induction of several endothelial stem and/or progenitor cell markers, including *Procr, Angpt2*, and *Cd34,* as well as the angiogenic marker *Apln*. Five days post-injury, another general capillary subtype emerged, characterized by a pro-proliferative gene expression signature (e.g., *Mki67, Aplnr*, and *Foxm1*) [70]. Aged mice were characterized by reduced pulmonary expression of *Apln* in an independent single-cell atlas of young and aged animals [71], and older mice were characterized by failed resolution of (diphtheria toxin induced) lung injury and excessive mortality, in an extent similar to young mice treated with an *APLNR* antagonist [70]. Although this would need to be corroborated in a human setting, specialized pulmonary capillaries and the APLN-APLNR signaling axis may thus play a pivotal role in maintaining a healthy, young status of the lung (and potentially brain, see above) endothelium.

#### Healthy aging

Dedicated single-cell resolution profiling of young versus aged healthy lungs has started to reveal novel insights into endothelial features of the healthy aging lung (Fig. 1, Table 1). A multi-omics lung atlas combined single-cell transcriptomics with whole proteome analysis in young and old mouse lung tissue [71]. Aging led to an overall increased state of transcriptional noise in most cell types, including vascular ECs. In addition, pro-inflammatory genes, such as *Il1b*, were differentially expressed in capillary ECs of healthy aging mice [71]. Along those lines, another murine cell atlas displayed aging changes in healthy young and old mice in lung, kidney, and spleen, and revealed an upregulation of IFN-related genes in aged lung and renal EC populations [72]. Similarly, genes related to the senescence-associated secretory phenotype (SASP; see Box 1) were found to be enriched in highly abundant capillary ECs in lung tissue of aged primates. Aging led to an overall inflammatory state and to increased interactions between ECs and immune cells, and included enhanced expression of *IL7* and the hypoxia-induced, pro-inflammatory marker *NFKB1* [73]. Furthermore, general capillaries in mice were shown to become specifically enriched for *Vwf,* a marker associated with EC-dysfunction, upon aging [67].

#### Aging-associated disease

The pro-inflammatory effect of aging on healthy lung ECs can be confirmed in scRNA-seq analysis using a fast-aging mouse model of Hutchinson-Gilford progeria, where upregulation of inflammation-related pathways in lung ECs resulted in a potential systemic effect [74]. From a human point-of-view, while extensive atlasing efforts [9, 75, 76] allowed for an all-encompassing overview of endothelial populations in healthy human lung tissue, a thorough interrogation of how aging affects these is still lacking. However, multiple studies analyzed age-related pulmonary diseases, such as IPF (idiopathic pulmonary fibrosis), COPD (chronic obstructive pulmonary disorder) and COVID-19 [7, 66, 77, 78], and helped reveal EC phenotypes and gene expression signatures that may be at play in healthy aging as well. For instance, in human lung tissue from IPF, smokers, and COPD patients, a specialized systemic/peribronchial EC population was found, defined by the marker gene *COL15A1*, which was enriched in IPF lungs around fibrotic areas [66]. This finding could be recapitulated in analysis of a cohort of lung tissues from IPF and COVID-19 patients [7]. This study, in which most patients were of older age, also observed a loss of general capillary and aerocyte ECs in both age-related diseases compared to healthy control tissue, as well as an increase of venous EC populations. Similar observations were made in drug-induced IPF in mice, where the influence of age on disease outcome was observable by reduced aerocyte abundance, an increase of venous ECs, and remodeling of the vasculature in 18-month old mice, stressing the impact of ECs in this condition [77]. In fibrotic conditions (COVID-19, IPF), an enrichment of genes involved in cellular stress, as well as signatures suggestive of hampered immunoregulation and loss of vessel wall integrity were furthermore found [7]. Whereas the uncoupling of disease versus aging-associated EC features is difficult, the EC alterations observed in these studies may hint toward the types of EC subpopulations and gene expression signatures at play in healthy aging of the human lung.

### Heart and associated vasculature

Cardiac ECs are an important player in heart physiology and pathophysiology. One of the first comprehensive single-cell atlases of healthy human heart tissue detected a heterogeneous range of EC subtypes spanning the entire cardiac vascular bed, including an immunoregulatory capillary EC subpopulation, as well as arterial ECs from the atrium [79]. From an aging point-of-view, an increasing amount of single-cell resolution studies have been conducted in the heart over the past few years, and have helped to substantially increase our understanding of specific changes in the cardiac endothelium in healthy and pathological aging (Fig. 1, Table 1).

#### Healthy aging

In mice, single-cell profiling studies revealed that coronary artery cells were among the top-ranking cell types most susceptible to aging-associated changes [80], and that ECs were predicted to acquire the most senescent phenotype, in comparison to other cardiac cell types [81]. The latter finding was corroborated in primate heart single-cell data [73]. Furthermore, scRNA-seq performed on aortas and coronary arteries from young and old healthy cynomolgus monkeys [82] revealed and upregulation of atherosclerosis-related pathways (e.g., calcium signaling, lipid response, inflammation) in aged EC subtypes in both vasculatures. Commonly downregulated processes included ECM organization and angiogenesis. Each vasculature also exhibited specific responses to aging: aortic arches were more sensitive to coagulation and external stimuli, while coronary arteries were prone to infection and leukocyte activation. Increased transcriptomic noise was also prominent in vascular ECs and fibroblasts during primate aging. Lastly, pro-inflammatory *IL7* was found upregulated, and *FOXO3A,* a longevity gene and regulator of vascular homeostasis, was downregulated in the majority of vascular subpopulations in old monkeys [73, 82].

A study focusing on the neuro-vascular interface of the myocardium showed a decrease in microvascular density in 22 month-old mice (compared to their young counterparts), and RNA-sequencing showed that pathways associated with neuronal impairment were upregulated in cardiac ECs from old mice [81]. One of the upregulated genes from those pathways was *Sema3a*, which could also be confirmed in an in vitro model of induced senescent ECs from aged mouse hearts. Different models of premature senescence furthermore indicated that the induction of senescence in the endothelium suffices to induce sympathetic denervation in the heart, stressing the influence of senescent ECs on the interplay of the nervous system and cardiac vasculature during aging, and showcasing a compelling example of how aging-associated findings from single-cell level data can lead to intriguing functional results [81].

Only recently have single-cell studies focused on the human heart tissue in different age settings, albeit mainly in disease settings. In human cardiac tissue after heart failure, as well as in healthy controls across age and sex, regression analysis between healthy young and old donors revealed several aging-associated genes differentially expressed in EC populations (e.g., *SLC22A23, CMTM4, KCNK6* (up), *PROM2, RBP1, COPS4, CCDC28B* (down)). However, significant enrichment of particular pathways or molecular processes among the age-associated EC genes were not reported, and an overall shift of EC populations could also not be observed in healthy donors between age groups [83]. Lastly, focusing on heart tissue from healthy aged donors, a recent study exploiting single-nuclei transcriptome and chromatin accessibility profiling revealed IFN alpha and gamma related genes and altered accessibility of genes associated with the NF-κB pathway to be upregulated in vascular ECs upon aging. This study is however limited in terms of age range, as only one donor was under the age of 43 [84]. While aging-associated phenotypes of the healthy human cardiac endothelium are thus starting to be uncovered, future studies at larger scale are required to obtain a more robust understanding of their molecular underpinnings and translational potential.

#### Aging-associated disease

As we age, a decline in the structure and function of the heart results in increased susceptibility to myocardial infarction, ischemic cardiomyopathies, and heart failure. In a mouse model of myocardial infarction, scRNA-seq revealed an increase in inflammatory markers in late stages post-injury. A transient mesenchymal transition of ECs was also found, but in earlier stages after injury [85]. Similar trends were observed in biopsies of the human right atrium in the context of heart failure and myocardial infarction [86]. Here, suspension and spatial transcriptomics indicated evidence of microvascular dysfunction in early stages of disease, signatures of inflammation throughout all disease stages, and signatures of hypertrophic signaling in late disease stages. Whereas the persistent nature of hypertrophic signaling is accompanied by a more permanent mesenchymal transition of ECs remains to be elucidated. Expression of the mechano-activated transcription factor *KLF2* was reduced in the majority of ECs in ischemic heart tissues, which may represent a possible driver of endothelial dysfunction in this condition [86]. Capillary ECs were found most susceptible to gene expression changes in a cohort of dilated (nonischemic) cardiomyopathy versus control donors [83]. Angiogenic regulators (e.g., *VEGFA, VEGFC, APLNR, FGFR1*) were furthermore enriched in cardiomyopathy-derived vascular ECs [83]. In an independent study, increased fractions of lymphatic and angiogenic EC subtypes were found in patients with ischemic cardiomyopathy [87]. Similarly, an increased abundance of lymphatic and angiogenic EC subtypes was also observed in heart tissue derived from hypertrophic cardiomyopathy patients [88]. While increased inflammatory gene signatures thus appear to be a common finding in healthy and pathological aging of the cardiac endothelium, elevated angiogenic signaling seems to be specific to aging-associated heart disease (and in contrast with the generally decreased angiogenic signals observed in healthy aging (Fig. 1, Table 1)).

### Skin

ECs of the vascular and the lymphatic systems are located in the dermis layer of the skin [89]. The skin harbors specialized EC populations, tailored to the gatekeeper/barrier function of this tissue. For example, certain capillary populations in the skin are characterized by high expression of HLA-II and therefore suggested to facilitate immune cross-talk [90]. In line with this, skin-specific lymphatic and vascular EC populations were found to regulate and recruit immune cells, by regulating the transmigration of cells into the dermis or in adjacent lymph nodes. More specifically, a vascular EC subpopulation characterized by selective expression of *ACKR1* and additional inflammatory cytokines, chemokines, and leukocyte adhesion molecules was found to be upregulated in inflammatory skin diseases [91]. While tempting to speculate that those pro-inflammatory EC populations may also play a role in skin aging, this remains to be elucidated. In general, little is known about the role of specific EC subpopulations in the skin in the context of (healthy) aging. However, it is well-known that the aging skin microvasculature becomes reduced, impaired or stiffened, resulting in reduced wound healing [92].

#### Healthy aging

While still sparse, scRNA-seq studies of the aged skin are starting to shed new light on these phenomena (Fig. 1, Table 1). A detailed single-cell atlas on human skin aging used eyelid skin biopsies from healthy young, middle-aged and aged donors, and confirmed an overall decrease of ECs in the aging dermis [93]. On a transcriptional level, DNA repair-related pathways were downregulated, while the transcriptional noise of ECs and fibroblasts increased considerably compared to other cell types. Differential regulation of EC-fibroblast interactions could also be detected, as pro-angiogenic interactions involving NOTCH3-HES1 signaling decreased with age [93]. Ichijo and colleagues independently confirmed the latter finding, by revealing a loss of angiogenic cross-talk between ECs and fibroblasts, accompanied by increased expression/secretion of the anti-angiogenic *Ptx3* by fibroblasts, altogether suggested to contribute to dermal stiffening and vascular atrophy [94]. Future studies in independent donors, alternative parts of the skin, and/or in aging-associated dermal conditions are expected to further complement these findings.

### Female reproductive system (Ovary, Uterus, Myometrium)

The female reproductive system (FRS) poses a unique and specialized setting for the study of endothelial aging. Not only does the vascular network of the ovarium regularly and uniquely undergo angiogenesis throughout adult life [95], but pre- and post-menopause settings moreover offer a particularly interesting approach to study tissue aging. Concerning the latter, it is known that decreasing estrogen levels after menopause can influence EC function by reducing endothelial nitric oxide synthase (eNOS) levels, leading to overall impairment of the vasculature [96].

#### Healthy aging

A single-cell atlas in primates analyzed ovarian aging and detected several DEGs in the EC subcluster. Aging-enriched genes were related (but not limited) to immunoregulation, cellular stress responses, and proliferation/cell survival (e.g., *CD74, FOS, FOSB, MYC, EGR1*) [97]. Among genes decreased in the aging ovarian endothelium was the capillary marker *RGCC* [97], possibly indicating a decreased microvascular network. However, this is a postulation that requires further investigation and study, as no difference in vascular density and stability could be observed by high-resolution imaging in the uterus of old mice [39].

Focusing on fallopian tube and ovaries after menopause from human healthy donors, a recent study detected multiple, distinct EC subclusters using single-cell profiling. Analysis of single-cell Assay for Transposase-Accessible Chromatin (scATAC)-seq data revealed an enrichment of SOX family TF activity in ECs from the fallopian tube. While temporal changes remain unclear due to lack of young or pre-menopausal samples [98], TFs of the SOX family have been shown to be strongly dysregulated in the aging endothelium of various mouse tissues [56]. Healthy-aging analyses of the ovary and myometrium at single-cell resolution revealed a loss of lymphatic EC density and an overall decrease of blood vascular ECs in aged samples [99, 100]. Furthermore, EC clusters revealed a decrease in genes related to the proteasome, DNA repair, and metabolism, as well as an increased expression of genes linked to apoptosis, senescence, cell-cycle, and immunoregulation in aged donors. Angiogenesis-related findings were conflicting, as signatures related to VEGF signaling were found increased in aged donors in one study [99], while findings of decreased EC abundance in myometrial aging, and decreased HGF, ANGPTL, and EDN pathway signaling between stromal cells and ECs would suggest impairment of angiogenesis in the aging myometrium [100]. In line with findings in the aging muscle, the number of interactions between ECs and other cell types in aged samples decreased [99]. Finally, a tissue-specific contractile capillary EC population was found in a comparative myometrium atlas of healthy human donors pre- and post-menopause [100]. Upon aging, a decrease in function and size of this population could be observed on a spatial level, as well as a decrease in their marker genes *FLNA* and *ACTG2*. In addition, the angiopoietin-like pathway was dysregulated in ECs of the aged myometrium, all suggestive of alterations to regulation of blood flow and hampered vascularization [100]. Unfortunately, while additional in-depth single-cell studies are available in context of the aging FRS, an analysis of the endothelium is typically lacking [97, 101]. The endothelium in FRS-associated diseases pre- and post-menopause has also not been profiled at single-cell resolution yet, precluding a more robust interrogation of EC heterogeneity over the course of FRS aging in health and disease.

### Kidney

The kidney is one of the most vascularized organs in the human body, in which renal ECs play a key role in the regulation of blood flow, vascular permeability, coagulation, and immune responses [102]. During aging, the density of renal microvessels and capillary pericytes are decreased [103]. Moreover, age-induced alterations of ECs, such as decreased eNos and increased Et-1 expression, lead to an impaired regulation of vascular tone [104–106]. scRNA-seq has revealed a remarkably degree of heterogeneous ECs, which can be classified into ECs belonging to the glomeruli, as well as the renal arteries, capillaries, and veins [4, 107–109].

#### Healthy aging

Single-cell analyses in young and aged mice revealed a reduction in capillary ECs in the aged kidney, suggestively linked to an impaired glomerular filtration rate [80]. Independent single-cell resolution studies furthermore revealed several age-related transcriptional changes in the renal endothelium and its distinctive EC subtypes. Aged kidney EC populations were for instance characterized by increased expression of genes associated with IFN signaling [72]. Moreover, compared to other EC subtypes, glomerular ECs had the largest number of age-induced and senescence-associated DEGs [110]. Glomerular and capillary ECs were furthermore found to exhibit an upregulation of genes related to inflammation, such as *Cxcl1, Cxcl10,* and *Sgk1*. Notably, *Serpine1* (also known as PAI-1), described as a potential mediator of senescent EC-induced podocyte loss, and associated with multiple age-related kidney diseases [111], was identified as a highly upregulated gene in aged glomerular ECs [110]. Additional genes related to kidney dysfunction (e.g., *Fstl1, Edn1, Il6*, and *Aqp1*) were also upregulated upon aging, suggesting the aged renal endothelium may be characterized by an increased susceptibility for kidney diseases [110]. As single-cell atlases of ECs in aging-associated renal disease are still lacking, commonalities and differences between the aged renal endothelium in healthy and disease conditions await further exploration and validation.

### Spatial and functional evidence of aging-associated vascular heterogeneity

Despite the wealth of information already gained by atlasing efforts from an EC-aging perspective, single-cell studies typically fall short in comprehensive functional validation of major findings. Moreover, ECs are surrounded by a wide range of diverse cell types, with which they spatially and temporally communicate in a highly organized fashion. Exposing such intricate spatial architectures and landscapes will enable us to understand how ECs communicate with each other and their environment, and how the magnitude and breath of these interactions change during lifespan. Spatial analysis of gene and protein expression will be imperative to reach this goal, but has not been heavily exploited for the study of vascular heterogeneity and aging yet. In the following paragraph, we therefore provide an overview of our current understanding of aging-associated endothelial and vascular heterogeneity across tissues, from both an in situ/spatial and functional perspective, derived using approaches that offer a valuable, high-resolution complement to single-cell omics.

### Heterogeneity of the tissue vasculature in space and time

Using high-resolution 3D imaging in mice, declines in arterial density and capillary abundance, accompanied with a loss of PDGFRβ^+^ pericytes, were identified as a key characteristic of aging in various organs (e.g., brain, heart, kidney, spleen, thymus) [39]. This aligns with the recurring observation of decreased (micro)vascular abundance in single-cell atlases of aged tissues as mentioned above. Conversely, tissues with high regeneration potential (e.g., intestines, uterus, skin) preserved their vascular network throughout lifespan [39]. The liver, considered another highly regenerative tissue, did however not retain its vasculature in mice, while the human liver maintained vascular density upon aging [39], suggesting potential species-specific differences in aging-associated vascular attrition. Independent histological studies in mice confirmed capillary loss in the aged brain and heart, as well as capillary and pericyte loss in the aging kidney [40, 81, 112–114]. Similar findings were partially observed in humans, with imaging-based studies revealing decreased vessel density and pericyte numbers in the aged kidney, muscle, and spleen, while the aged skin preserved its vasculature [39, 115]. Examination of post-mortem brain tissues from young and older individuals furthermore demonstrated a significant reduction in pericyte coverage in aged individuals [116]. These findings indicate that age-related changes in vascular density are tissue-specific, and that the extent of aging-associated vascular attrition may (at least to some extent) be linked to the regenerative capacities of a tissue.

### Diversity in endothelial susceptibility to aging-associated stressors and hallmarks

**Fig. 2:**
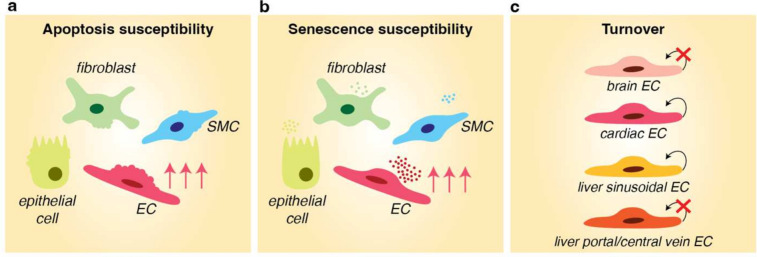
Heterogeneity in susceptibility of ECs to aging-associated stressors and responses. **a** ECs in numerous tissues (e.g., liver, heart, muscle, lung) are more prone to undergoing apoptosis, induced by various stressors (e.g., therapy-induced toxicity, aging), in comparison to other cell types. **b** ECs in several aging mouse tissues (e.g., heart, kidney, liver) have been shown to be more prone to acquiring a senescent gene expression signature in comparison to other cell types, as illustrated by a more prominent senescence-associated (secretory) phenotype in ECs. **c** Heterogeneous EC turnover rates during lifespan: the majority of ECs in the brain neurovascular unit of mice do not exhibit signs of turnover throughout lifespan, whereas a proportion of cardiac ECs (human) have been predicted to renew annually, without signs of decline with advancing age. In mice, hepatic ECs lining the portal and central vein were shown to not divide or turnover, while sinusoidal ECs show signs of active division/turnover during the animals’ lifespan. EC = endothelial cell; SMC = smooth muscle cell

Given their continuous exposure to potentially damaging stimuli (e.g., hemodynamic forces, inflammatory cytokines, reactive oxygen species derived from tissues and/or circulating immune cells), ECs are believed to be particularly prone to typical hallmarks of aging, including cellular senescence (Box 1) [117]. The abovementioned heterogeneous nature of microvascular density across aging organs furthermore suggests a potential differential sensitivity of specific vascular beds to such aging-associated phenotypes. In line with this, the aging-related vascular decline in some murine organs could at least in part be explained by increased EC apoptosis (muscle, thymus). In some other tissues (kidney, spleen) this was not the case [39], suggesting that in general there appears to be heterogeneity in apoptotic priming of ECs across organs and tissues. Moreover, a scRNA-seq analysis of a wide range of cell types and tissues revealed that ECs in liver as well as mesenteric or gonadal adipose tissue were more prone to transcriptional changes in response to accelerated aging (via heterochronic parabiosis) compared to other tissue ECs (e.g., muscle, trachea, diaphragm) [118]. The authors speculate that, at least the adipose tissue findings, may be related to the fact that in an independent study this tissue was shown to present with aging-associated transcriptomic changes already in mid-life, before such changes typically appear in most other mouse tissues [60]. In vivo BH3 profiling in key murine organs from birth to adulthood revealed that the mitochondria of ECs were particularly sensitive to anti-cancer therapy-induced toxicity, as evidenced by increased apoptotic priming [119]. Most other non-vascular cell types in the liver, heart, and lung did not show such increased apoptotic sensitivity [119] (Fig. 2a). In an independent study, analysis of the gastrocnemius muscle in aging mice revealed a substantial increase in apoptosis of capillary ECs, to a much larger extent than other stromal cell types or myofibers [120]. Moreover, ECs in the aged mouse heart were shown to most dominantly acquire a senescent gene expression signature in comparison to other cell types (e.g., cardiomyocytes, fibroblasts) [81]. Across mouse organs, the liver was among the first tissues to accumulate senescent cells, and the majority of these cells was found in the sinusoidal microvasculature. Targeted removal of senescent sinusoidal ECs was however detrimental to liver function and overall health of the animal [121]. Lastly, in the aging mouse kidney a specific increase in endothelial senescence was observed, which induced apoptosis in podocytes via secretion of PAI-1 [122]. Altogether, these findings are in line with the concept of specific priming of ECs toward aging-associated features of apoptosis and senescence, in a heterogeneous fashion across tissues (Fig. 2a, b).

### Heterogeneous EC turnover rates during lifespan

The increased susceptibility of ECs to apoptosis and senescence may possibly be related to increased vascular attrition observed in a wide range of aging tissues. However, isotope tracing studies have revealed further complexity by showing variations in EC turnover during lifetime (Fig. 2c). In all organs, blood vessels consist of a mixture of ECs, perivascular cells, and extracellular components with varying lifespans. In the brain, the majority of ECs in the neurovascular unit of mice do not exhibit signs of turnover, suggesting they are as old as the animal itself, except for occasional capillary ECs [123]. Hepatic ECs lining the portal and central vein do not divide or turnover either, while, on the other hand, the microvascular sinusoidal bed and stellate cells in the liver show signs of active division/turnover during the animals’ life [123]. As the sinusoidal microvasculature seems to be prone to senescence and tends to be affected in aging-associated pathologies of the liver [121, 124–126], it is interesting to speculate that specific sinusoidal EC turnover rates may play a role in these phenomena. In the human heart, a modest decline in the number of ECs was observed during adulthood and aging, and ECs were shown to have the highest exchange rate compared to other cardiac cell types [127]. Mathematical modeling predicted that approximately 16.7% of cardiac ECs are renewed annually in the adult heart, and this rate does not seem to drastically decline with age [127]. If and how such turnover rates relate to aging (disease) phenotypes of the heart, and whether these observations can be extended to other tissues, remains to be elucidated.

## Discussion & future directions

Over the years, single-cell resolution studies have started to shed light on how the gene expression landscape of the heterogeneous, tissue-specific endothelium responds to the process of aging. Independent studies in different tissues and organs commonly revealed a phenomenon of decreased expression of microvascular and angiogenic gene signatures, and the reduced abundance of (capillary/microvascular) ECs (Fig. 1, Table 1). This finding was confirmed in a recent scRNA-seq atlas of murine cell types across tissues, ages, and conditions [128], and by numerous independent histology-based efforts. Increased expression of inflammation and immunoregulatory marker genes furthermore characterizes the aging endothelium across many tissues (Fig. 1, Table 1). The skin, a tissue with high regenerative potential and absence of overt capillary rarefaction upon aging, was shown to harbor less prominent EC-selective enrichment of inflammatory gene expression compared to the endothelium in other aging tissues [39]. While further research is required, an intriguing association between the extent of inflammatory pathway induction and capillary rarefaction may thus exist. Pan-tissue atlasing efforts, in which high quantities of ECs are profiled and compared jointly across organs, will be required to systematically pursue this hypothesis, ideally in combination with animal/donor matched vascular histology analyses.

While the number of tissue-specific atlases detecting/including ECs at sufficient quantifies in their datasets is steadily increasing, an EC-centered analysis or discussion of their relevance in aging(-associated disease) is not infrequently lacking to date [76, 89, 97, 101, 129, 130]. Nevertheless, the expanding body of single-cell resolution data on young and aged ECs offers exciting opportunities for future integrative meta-analysis efforts toward tackling the currently unmet need of defining robust transcriptional patterns of healthy EC aging within and across (human) tissues. Certain limitations of single-cell transcriptomics may however need to be taken into account. Challenges of integrating datasets of ECs profiled in different laboratories, using different isolation protocols, library preparation methods and sequencing platforms (as discussed in [10]) need to be considered. Moreover, mixed perceptions about the increased levels of transcriptional noise, and to what extent this may influence the make-up of aging-associated EC gene expression fingerprints, need to be acknowledged and investigated [131, 132]. Additionally, the age of an organ’s vascular bed has thus far typically been linked directly to the chronological age of the organism. Less attention has been given to the age of the constituent ECs that make up the organ’s vascular network, which, as described above, may not reflect the chronological age of the organism. In other words, we cannot simply assume a 1:1 correlation between the age of individual (tissue-specific) ECs and the age of the organism. One might thus wonder whether regenerative ECs (i.e., ECs showing active turnover during lifespan) are differentially susceptible to aging-related stresses compared to their non-regenerative counterparts? Exploring this question, for instance in different cardiac vascular beds with potentially diverse turnover rates, can have significant fundamental and clinical implications related to aging-associated cardiovascular disease. Additionally, investigating the molecular mechanisms that decouple the organism's chronological age from the biological age and turnover rate of individual cell types, including ECs (for instance by generation of tissue- and cell-type specific aging clocks [133, 134]), will greatly enhance our understanding of how the aging tissue microenvironment is formed and maintained.

Notwithstanding these challenges and limitations, the unraveling of aging-associated EC heterogeneity using single-cell technologies holds undeniable promise for the discovery of interesting novel, specialized EC subtypes and gene expression signatures with a potential key role in vascular aging. Exciting prospects and ambitions are arising in light of selective targeting of the endothelium and specific EC subtypes [10, 11], together with progress in deciphering the potential of detecting and therapeutically targeting aged (senescent) ECs (reviewed in [135]). Consequently, studies aimed at translating EC-derived single-cell omics data into clinically interesting and feasible follow-up studies to recover or rejuvenate the aged endothelium are expected to populate the landscape of vascular aging research in the near future.

Box 1 Endothelial cell senescence Exposure to damaging stimuli and stressors results in (among others) DNA damage, telomere shortening, mitochondrial dysfunction, chromatin changes, and alterations in energy sensor pathways. Changes induced by these stimuli lead to the activation of tumor suppressor pathways, such as p53–p21 and p16-RB, resulting in cellular growth arrest and senescence [137, 138].

The senescence-associated secretory phenotype (SASP) is a common characteristic in senescent cells, and canonical SASP factors include inflammatory chemokines, cytokines, extracellular matrix proteases, growth factors, bioactive lipids, and reactive oxygen species (ROS) [139]. Senescent ECs can secrete elevated levels of ROS, pro-inflammatory cytokines/chemokines (e.g., IL-1β, IL-6, IL-8, CXCL11, PAI-1) and adhesion molecules (e.g., VCAM-1, ICAM-1), and pro-fibrotic cytokines (e.g., Endothelin-1). Moreover, they are characterized by altered expression of vasoactive mediators (e.g., nitric oxide) [117, 140]. Senescent ECs also suffer impaired angiogenic potential [135, 141] and undergo several metabolic changes, including alterations in the renin/angiotensin system (RAS), active aerobic glycolysis, decreased pyruvate dehydrogenase kinase activity, disruption of serine synthesis and of pentose phosphate pathway activity, and uncoupling of eNOS [135]. Typical inflammation- and oxidative stress-associated changes with vascular aging are thus believed to be linked to an increase in the number of senescent ECs [117]. In addition, senescent ECs are enlarged, flattened, and unable to properly align in response to shear stress [117].

On the protein level, senescent cells (including ECs) can generally be identified by numerous markers, including senescence-associated beta-galactosidase (SA-beta-gal), increased levels of p15, p16, p21, p53, and/or ADP-ribosylation factor, measurements of telomere shortening, the DNA double-strand-break marker γH2AX, and senescence-associated heterochromatin foci [142]. Cell-cell heterogeneity in the susceptibility and presentation of senescence complicates a unified detection of senescent cells via gene expression analysis. However, various meta-analyses have contributed senescence-associated gene sets that can be exploited for analysis in the context of specific tissues and cell types, including ECs [143–147].

Senescent ECs and their secreted factors are major contributors to vascular dysfunction and the pathophysiology of various cardiometabolic diseases, as reviewed in [117, 135, 140]. Emerging evidence suggests that targeting senescent ECs with senolytics can be an effective strategy to suppress cardiometabolic diseases, although results are still conflicting [135, 140]. In addition, adverse effects of senolytic therapy or targeted removal of p16^high^ senescent cells on vascular and overall tissue/organism health and function have been reported as well [121, 148], stressing the need for extensive validation and follow-up.
